# Special Issue “Computer-Aided Drug Discovery and Treatment”

**DOI:** 10.3390/ijms25052683

**Published:** 2024-02-26

**Authors:** Yaron Ilan

**Affiliations:** Department of Medicine, Hadassah Medical Center, Faculty of Medicine, Hebrew University, Jerusalem 91120, Israel; ilan@hadassah.org.il

This Special Issue aims to highlight some of the latest developments in drug discovery. Computer-Aided Drug Discovery and Treatment (CADDT) is a multidisciplinary approach that combines bioinformatics, chemoinformatics, structural biology, and artificial intelligence (AI) [1]. CADDT has proven to be instrumental in navigating diseases’ intricate landscapes by facilitating drug development, optimizing treatment strategies, and improving the patients’ outcomes [2].

CADDT is fundamentally based on virtual screening [3]. The molecular binding affinity is predicted using computational models of target proteins. Drug discovery traditionally involves synthesizing and testing many chemical compounds, which is a time-consuming and resource-intensive process. Through virtual screening, researchers can computationally evaluate potential drug candidates, prioritizing those that are likely to exhibit the desired therapeutic effects. Reducing the number of compounds that need to be synthesized and experimentally validated expedites the drug discovery timeline. CADDT allows the design of molecules with specific properties that make them effective against a target protein [4,5].

In structural biology and molecular dynamics simulations, X-ray crystallography, NMR spectroscopy, and cryo-electron microscopy are essential for determining the biomolecule’s three-dimensional structures [6]. The structures serve as a basis for understanding how the drugs interact with their target proteins, facilitating the development of more effective and selective therapeutics. We can better understand the biological molecules’ dynamic behavior by simulating molecular dynamics. Through the computational modeling of atoms and molecules’ movements over time, the proteins’ flexibility and conformational changes provide insights into the drug molecules’ binding mechanisms to optimize drug designs and predict drug–protein interactions under various conditions [7].

The interdisciplinary field of chemoinformatics, which combines chemistry and informatics, plays a critical role in CADDT [8]. Computational methods are used to analyze chemical data, extract meaningful patterns, and predict chemical properties. They are used to identify potential drug candidates for virtual screening, similarity searches, and chemical database analysis [3]. As part of chemoinformatics, ligand-based drug design creates new molecules based on existing ligands. A quantitative structure–activity relationship (QSAR) model enables correlation between the compounds’ chemical structure and biological activity, which can be used to design new molecules with greater potency and selectivity [9].

CADDT is being enhanced using artificial intelligence and machine learning [3]. They can extract knowledge and predict drug discovery and treatment outcomes by analyzing vast datasets. They can identify challenging patterns by analyzing complex biological and chemical data. They allow one to predict drug–drug interactions, analyze patient-specific data, and suggest novel drug targets based on large-scale biological and chemical information. By continuously exposing the models to more data, they become more accurate, identifying new drug candidates and optimizing individualized treatment strategies [10].

CADDT can optimize the pharmacokinetics and pharmacodynamics of drugs by providing information about their absorption, distribution, metabolism, and excretion in the body [11,12]. It is crucial to have this knowledge to design drug formulations that have enhanced efficacy, minimize the side effects, and improve patients’ compliance. Incorporating PK/PD models allows researchers to simulate different dosing regimens, predict drug concentrations at target sites, and optimize treatment protocols for the maximum therapeutic benefit. The existing therapeutics are rendered more effective by refining their administration strategies [13].

A CADDT approach contributes to personalized medicine and patient-specific treatment. By analyzing genetic variations, lifestyle factors, and other patient-specific data, CADDT can identify optimal drug regimens that are likely to be effective with minimal side effects [14]. A predictive model is used in CADDT to identify the patients that are more likely to respond positively to a particular treatment. By stratifying these patients according to their molecular profiles, more targeted and personalized treatments can be delivered [15].

Using Constrained Disorder Principle (CDP)-based systems to improve CADDT: Second-generation AI systems that use CDP-based variability signatures improve CADDT’s accuracy and personalize its output [16]. CDP-based systems account for the biological noise that characterizes all biological systems. These systems focus on the patients’ clinical outcomes as an output for the algorithms and continuously implement signs of biological variability into the algorithm to improve drugs’ efficacy. These systems improve the response to chronic medications, overcome drug tolerance, and reduce the number of side effects, thereby improving patients’ adherence [17]. The option for CDP-based drug design was suggested to develop a variability-based molecular structure.

Ethical Considerations and Challenges: The accuracy of computational models, the integration of several complex data sources, and the validity of virtual predictions through experimental means remain ongoing concerns [18,19]. Computational models are only as reliable as the data used to train them are, which can introduce biases and limitations. In addition to data privacy, transparency, and responsible AI use in healthcare, the ethical considerations must be carefully considered [20].

In summary, CADDT incorporates computational methods and cutting-edge structural biology technologies. By accelerating drug discovery, it improves the personalization and effectiveness of treatments. In recent years, the approach to drug development has been transformed by virtual screening, rational drug design, structural biology, chemoinformatics, and artificial intelligence, which optimize the identification of potential drug candidates and the creation of treatment strategies tailored to each patient. Emerging immersive technologies and quantum computing may further improve the capabilities and accuracy of CADDT.
